# Ethnomedicine and ethnobotany of fright, a Caribbean culture-bound psychiatric syndrome

**DOI:** 10.1186/1746-4269-6-9

**Published:** 2010-02-17

**Authors:** Marsha B Quinlan

**Affiliations:** 1 Department of Anthropology, Washington State University, Pullman, WA 99163, USA

## Abstract

**Background:**

"Fright" is an English-speaking Caribbean idiom for an illness, or ethnomedical syndrome, of persistent distress. A parallel ethnopsychiatric idiom exists in the French Antilles as sésisma. Fright is distinct from susto among Hispanics, though both develop in the wake of traumatic events. West Indian ethnophysiology (ethnoanatomy) theorizes that an overload of stressful emotions (fear, panic, anguish or worry) causes a cold humoral state in which blood coagulates causing prolonged distress and increased risks of other humorally cold illnesses.

**Methods:**

Qualitative data on local explanatory models and treatment of fright were collected using participant-observation, informal key informant interviews and a village health survey. Ethnobotanical and epidemiological data come from freelist (or "free-list") tasks, analyzed for salience, with nearly all adults (N = 112) of an eastern village in Dominica, and a village survey on medicinal plant recognition and use (N = 106).

**Results:**

Along with prayer and exercise, three herbs are salient fright treatments: Gossypium barbadense L., Lippia micromera Schauer, and, Plectranthus [Coleus] amboinicus [Loureiro] Sprengel. The survey indicated that 27% of village adults had medicated themselves for fright. Logistic regression of fright suffering onto demographic variables of age, education, gender, parental status and wealth measured in consumer goods found age to be the only significant predictor of having had fright. The probability of having (and medicating for) fright thus increases with every year.

**Conclusions:**

While sufferers are often uncomfortable recalling personal fright experiences, reporting use of medicinal plants is less problematic. Inquiry on fright medical ethnobotany (or phytotherapies) serves as a proxy measurement for fright occurrence. Cross-cultural and ethnopharmacology literature on the medicinal plants suggests probable efficacy in accord with Dominican ethnomedical notions of fright. Further, the cultural salience and beliefs about these medicines may give these medications extra psychoneuroimmune (i.e. mind-body) benefits, or placebo-like effects, for this stress-related folk illness.

## Background

In Caribbean Creole English, "fright" not only indicates sudden fear or shock, but also panic, anguish, and worry [1]. Fright also is an idiom for a prolonged, distressed state blamed on an emotional overload of fear, shock, panic, grieving or anguish. Accounts of Anglophone and Francophone Caribbeans allude to fright as an illness [2-5]. Antillean French Creole speakers call it *sésisma*, also written *sézisman *[cf [6]] (from the French *saisissement*, translating to shock or sudden chill). The French Creole term, like the Creole English one, indicates both an emotion and the illness that the emotion may generate. The sufferer is said to "have fright" or to "be frightened."

Here I describe the ethnomedical syndrome of fright, or *sésisma*, in the Commonwealth of Dominica, an island-nation in the Lesser Antilles. Dominicans are bilingual in English and French Caribbean Creoles, and Dominican culture shares traits with both English and French-speaking Caribbean islands, where fright, or *sésisma *is familiar. I describe Dominican views of fright's etiology, ethnophysiology, ethnopharmacology, and the epidemiology of fright in terms of its salience, treatment and occurrence in the Dominican village of Bwa Mawego.

Caribbean fright illness has not been previously detailed in the literature. Caribbean fright's perceived causes and symptoms differ from other fright illnesses from around the world (including *susto *among neighboring Hispanic populations). The general pattern of this ethnopsychiatric syndrome, or idiom of distress, is nevertheless reminiscent of others. In their seminal review *The Culture-Bound Syndromes*, Simons and Hughes [7] created a whole section on "the fright illness taxon," Simons and Hughes present seven taxa of syndromes that have culturally specific [or "bounded"] illness expressions, but with comparable etiology or symptoms.) Indeed, various world cultures associate an illness with emotional fright. Symptoms and treatments of fright illnesses vary from culture to culture, but all fright illnesses are blamed on a fright or trauma--many societies even use a term translating to "fright" for an illness. Other fright illnesses include, perhaps most famously, *susto *in Latin America [8], but also *ceeb *among Hmong [9], *fijac *in Yemen [10], *kesambet *in Bali [11], *lanti *in the Philippines [12], *mogo laya *in New Guinea [13], *narahati *in Iran [14], *saladera *in the Peruvian Amazon [7], and "reduced soul" in Cambodia [15]. Fright illnesses often include physical symptoms, psychological/behavioral symptoms, or a period of misfortune in the sufferer's life [7]. Those fright illnesses tend to share a local diagnosis involving soul loss: Distress potentially dislodges a sufferer's soul (or vital force), or scares the soul out of the body. Soul loss is *not*, however, part of the Anglo-Franco Caribbean fright explanation.

These Caribbeans blame fright not on soul loss but on physical changes in blood and nerves that occur in response to a trauma. Dominicans, like other Caribbean people, subscribe to a version of the New World hot/cold humoral system that has been documented throughout the New World, particularly in Latin America (for an overview, see [16]). Foster claims, in fact, that "humoral medicine in the Americas is the most completely described of all ethnomedical systems" [16]:2]. In the hot/cold humoral system, people group mental and physical states, plants, and animals into "hot" and "cold" categories. Here, "cold" or "hot" may refer to the temperature of air or bathing water, however "hot" and "cold" often refer to culturally ascribed symbolic values having nothing to do with thermal state. Health requires balancing hot and cold influences to an individual's body system [17]. Strong emotions charge the blood with humoral heat or cold; and frightful emotions are blood-chilling.

Cold blood leads to tense nerves. Sobo notes that Jamaicans regard nerves as anatomical (rather than mental) equipment, which is susceptible to malfunction [18]. Dominicans hold the same view, and they attribute malfunctions to "wear" on the nerves. Nerves wear through overuse but if one's blood is cold and soured by the fright, it exacerbates the rate and degree of nerve damage. Dominicans thus maintain that shock, fear, panic, or anguish and the resulting blood changes can leave a person in a state of constant stress, anxiety, or nervousness.

Villagers in Bwa Mawego use several herbal infusions to treat anxiety, or in their view, to hinder the cold humoral effects of frightful emotions. As they reckon fright to be extremely cold, they treat fright with herbs that they consider to be hot, or "heating." Ingesting the medicine thaws or warms the body toward the normal warm (neither too hot not too cold) state, at which nerves function best.

Dominicans recognize two types of fright. Most fright cases are the "short," "regular," "normal," fright, which is relatively acute, lasting around fourteen months or less. "Chronic fright," in contrast does not heal and ranges from reoccurring fright episodes to a continuous "frightened" state, which can be terminal. Though fright illnesses occur throughout the world, Franco/Anglo Caribbean frights particularly resembles syndromes in neighboring Hispanic populations. The term "fright" literally translates in Spanish to *susto. Susto *is a Latin American fright illness that also begins with a shock and includes symptoms of trembling, agitation, crying, difficulty sleeping, and general malaise [19]. These qualities overlap with Caribbean fright or *sésisma*, and *susto *shares some traits with other Latin American folk illnesses, *nervios *and *ataques de nervios *(see [20]). Caribbean fright illnesses also resemble Hispanic experiences of *ataques de nervios *("nerve attacks")(see [21]), which are panic attacks triggered by acute stress characterized by uncontrollable outbursts of shouting and crying, trembling, palpitations, and aggressiveness [22]. Finally, the Latin American concept of *nervios *("nerves"), which stems from social overburden and conflict and includes sadness, anger, sleep troubles, hopelessness (see [22-25]), seems not unlike Caribbean descriptions of the circumstances and symptoms of "chronic fright." Like the two Caribbean fright varieties, *ataques de nervios*, *susto*, and *nervios *share some etiologies and symptoms with posttraumatic stress disorder, anxiety and depression as recognized by the American Psychiatric Association (see [20,26,27]).

### Case study setting

The Commonwealth of Dominica is a small, island nation located between the French Departments of Guadeloupe to the North and Martinique to the South (15°N, 61°W). The island is mountainous, relatively undeveloped, and supports little agriculture or tourist industry compared to other Caribbean islands. Dominicans are bilingual in Creole English and French Creole. Dominica's population (approximately 68,000) is of mixed African, European (French and English) and Native American (Island-Carib) descent. Dominica is the last refuge of the Kalinago (Island-Caribs), and the only Native American reservation in the West Indies (Carib Reserve, a.k.a. Kalinago Territory) is there. All Dominicans, save some Kalinago, have ethnically mixed heritage, but frequency and intensity of Carib ethnicity wanes with distance form the ethnic center.

This research took place in Bwa Mawego, an east (windward) coast village near Kalinago Territory, where residents have mixed Afro-Caribbean and Kalinago heritage. The village's annual rainfall is between 100 and 150 inches per year, making for lush vegetation. The approximately 500 residents earn their living through subsistence gardening, fishing, and producing bananas and West Indian bay oil (a.k.a, bay rum, *Pimenta racemosa *[Miller] J.W. Moore), and some residents engage in wage labor. Almost everyone gardens, including those with other work. In addition to subsistence gardens at the village periphery, most land within the village is cultivated with fruit trees and other plantings, and families maintain small house-gardens for condiments and herbs for cooking and medicine.

Remote, even by Dominican standards, Bwa Mawego is located about a forty-minute drive from the main road, at the dead-end of a narrow, mountainous, and until recently often washed out road. Relative isolation reduces residents' economic opportunities. Even though increasing numbers of locals are high-school graduates and are getting jobs outside of the village [28], traditional ecological knowledge (TEK) remains the norm for dealing with subsistence and health.

The village's location also limits residents' access to outside biomedicine. There is a local health center that offers inoculations and a short supply of first aid materials and common medications (e.g., ibuprofen). The nearest pharmacy is a one-and-a-half to two-hour drive away. A doctor is available at the government health center 45 minutes drive from the village. Few villagers own private automobiles, however, and rides are expensive and sometimes difficult to arrange. Hence, all villagers rely heavily on traditional notions of illness and their corresponding home remedies--a system locally called "bush medicine."

Villagers assert over and over that everyone in the village is his own "bush doctor." Elsewhere in the Caribbean and in Dominica's capital town, there are herbalists who call themselves "bush doctors" and charge for their advice. In rural Dominican villages, which are largely kin-based, residents neither claim expertise (which would be immodest) nor charge their kin/neighbors for health advice. While some villagers know more, or are more interested in "bush medicine" than others, herbal advice is sought and given freely and with humility in tune with the generally egalitarian ethos of the village's horticultural roots [29]. Although the village of Bwa Mawego is modernized in several market-related respects (e.g. televisions and cellular phones are common) and the village is integrated into a larger society in some respects (e.g. through national elections), the village's isolation in the mountains and reliance on subsistence gardening results in day by day small-scale life-ways. As in many small scale societies, self treatment with traditional medicine is acceptable, accessible and common [30]). Self treatment, though easily overlooked, is the first resort and most common form of health care cross-culturally [31] and every society has a popular sector of medicine (sensu [31]), i.e., people self-treat and treat their dependants. Specialized practice of folk healers and health professionals (like any specialization or professionalization) appears as a factor of societal size and complexity, or with "the power of scale" [32]. As with most illness, recognition and diagnosis of fright occurs in the home. Treatment for fright is herbal and home-based as well.

Preparations of bush teas and salves in Dominica tend to be simple, often with one herbal ingredient, and are targeted at particular ailments [30]. This contrasts with traditions among other Caribbean peoples who use herbal mixtures as general tonics (e.g. as in the Dominican Republic, [33] and Cuba [34]). The Caribbean is an area of cultural variation with influences of indigenous peoples several colonial powers and African and Asian immigrants. Regional medical traditions likewise vary with some peoples using cure-all cocktails of various herbs and other groups targeting illnesses with single plants. Vandebroek et al. confirm that, "no detailed information exists in the published literature about the prevalence of these mixtures versus single-plant remedies in the ethnomedicinal traditions of Caribbean cultures or their migrant communities [33]." This paper reports case study data from one village that uses single plants and occasional simple blends, directed at particular illnesses. Qualitative interviews with individuals from other Dominican villages, including Kalinago Territory, thus far indicate similar treatments using single plants that target specific illnesses.

## Methods

Fieldwork for this project was conducted during eight trips to the study site between 1993 and 2008. Ethnographic data on local explanatory models and treatment of fright were collected using participant-observation, informal key informant interviews, a village health survey, semi-structured key informant interviews with bush medicine experts, freelist tasks with village adults, and an ethnobotanical and epidemiological survey.

### Informed Consent

Prior informed consent was obtained verbally at the time of each interview or for each field season working with key informants. Internal review boards of the University of Missouri and Washington State University examined and approved human subjects protocol for the protection of the study participants. The research followed ethical guidelines adopted by the American Anthropological Association [35] and the International Society of Ethnobiology Code of Ethics [36].

### Participant-observation and informal interviews

I used participant-observation (P-O) [37] to achieve qualitative understanding of the Dominican way of life and people's views, specifically those that deal with plants, illness and treatment. Opportunities for participant-observation in ethnobotanical activities and conversations abound in this subsistence gardening community. For example, while visiting with village residents I asked about their house gardens. I asked about planting procedures and names and uses of certain plants. I helped people with ethnobotanical chores such as garden work, peeling coffee and other food processing, brewing bush teas, and so forth. As I learned more about local medicine through general discussions, I began to focus my informal questions on health-related issues. I directed conversations so that people could recount their own health experiences and elaborate in detail on the circumstances surrounding illness events in their family and friends' life histories. P-O "sampling" is opportunistic, however, after years in the village, I have done some kind of participant-observation with at least half of the adults and many children in Bwa Mawego. Eventually, I conducted informal interviews specifically regarding fright. These were conversational and involved asking a representative sample of 30 village adults about their own direct and indirect experiences with and responses to fright events.

### Health survey

The health survey occurred in 1994 and involved asking every primary caregiver, usually a mother, a series of recall questions regarding the health of family members. These interviews were not directed specifically toward fright. Rather, I asked about the general health history and condition of all household residents. I asked them to recall any illness or injuries their family members had suffered in the past week, past month, and past year. Each time a woman mentioned an illness event, I asked her how the family member became sick to probe for the perceived etiology of the illness. I next asked what, if anything, anyone did to treat the sick person. If someone at home treated the sick person (which was usually the case), I asked the woman to describe the treatment. I also asked mothers who they sought out for bush medical advice and which villagers knew the most about bush medicine.

### Key consultant interviews

From the survey of mothers, five village residents stood out as particularly sought after for their bush medical advice. These five experts became key informants, or project consultants. They included three women, ages 39, 55, and 68, and two men, ages 25 and 49. Each consultant was interviewed three times during the 1994. The first interview was a long, general interview on the medical system including the kinds of health practitioners that villagers use under certain circumstances, local notions of ethnophysiology, and which illnesses the expert treated with bush medicines. During the second interview, I asked the experts which bush remedies they used for each sickness they listed during the previous interview. Next, I consulted with the experts on the use(s) of each bush medicine that he or she had listed. Finally, the consultants helped to gather samples of every remedy he or she had mentioned during the previous two interviews. The majority of the remedies were plants, for which voucher specimens were collected (see below). Data from these early key informants was foundational to the rest of the data-gathering. Years later, after analysis of quantitative data on illnesses and treatments, I returned to my key informants (during 2004-8 trips) to consult on fright specifically.

### Voucher Specimens and Identification

Key informants took me to find voucher specimens of each plant they had mentioned in previous interviews. I collected specimens on-site noting information about the plant and its growing conditions (see [38]:28-36). I repeated the process with multiple key informants to triangulate because some species have multiple local common names and some common names refer to multiple species. Dr. Steven Hill (Center for Biodiversity of the Illinois Natural History Survey) consulted on plant identifications and Dr. José Luis Fernández Alonso (Real Jardín Botánico, Madrid) consulted on the *Lippia micromera*. Vouchers are deposited at the University of Missouri Dunn-Palmer Herbarium (UMO).

### Freelists of illnesses

I conducted freelist interviews to obtain quantifiable data on the salience and intra-cultural variation of knowledge of illnesses and their treatments. In a freelist interview, an informant simply lists things in an emic category or "cultural domain" in whatever order they come to mind. The resulting list is a basic inventory of the items the informant knows within the domain [39,40]. The established ethnographic assumptions of the method are three-fold: First, individuals who know a lot about a subject list more terms than people who know less (geographic experts can list many countries [41]). Second, people tend to list terms in order of familiarity (people list the kin term "mother" before "aunt," and "aunt" before "great-aunt" [42]. And third, terms that most respondents mention indicate locally prominent items (Pennsylvanians [from the NE of the US] list "apple" and "birch" trees more frequently and earlier than they do "orange" or "palm" [43]).

Freelists are most efficient and accurate when the "domain" elicited is a narrow one (e.g. Indiana students inventoried more local birds when asked to list "backyard birds in Indiana" then when asked to list "birds you can think of") [44]. I thus conducted two successions of freelists to hone domains [45].

First, in June of 1998, with a quota sample of 30 adults stratified by age, sex and village location [see [29]] (approximately 1/4 of resident adults), I elicited the illnesses that villagers treat with "bush medicine." Those lists were analyzed for salience to find the bush-treated illnesses with greatest cognitive and cultural significance among the sample of respondents (table 1).

**Table 1:** Indicators of short-term and chronic varieties of fright, differences in bold print

	Short fright	Chronic fright
Etiology	**Single event **cause	**Successive events **cause

Symptom	Frequent recollections of traumatic event	Frequent recollections of traumatic event

Symptom	Difficulty concentrating	Difficulty concentrating

Symptom	Outbursts of anger/grief	Outbursts of anger/grief

Symptom	Persistent **arousal and hypersensitivity**	Persistent **dullness and sadness**

### Salience analysis of illnesses

Salience (or Smith's S, see [46]) is a statistic that accounts for an item's frequency of mention and is also weighted for list position (e.g., in the domain of English color terms, "red" is more salient--it appears more often and earlier in freelists--than "maroon"; [47]).

The first step in salience analysis is to calculate the salience rankings of items each individual freelisted.

Freelisted items in a subject's list are ranked inversely. If an individual lists 3 items, A, B, and C, in that order, then A = 3, B = 2, and C = 1. Each item's ranking is divided by the number of items listed, in this case 3, so that S (A) = 1, S(B) = .666, S(C) = .333.

The next step is to calculate the mean salience value, called composite salience (Composite S) for every listed item across all informants to reveal the intracultural salience of each item. Here, all subjects' salience scores for an item are summed and then divided by the number of informants in the sample (see [44,46]

Illnesses with the highest composite salience values are those that villagers most often treat with bush medicines. These common illnesses, or illnesses with the most emic importance in terms of home treatment, are the focus of my subsequent inquiry. Fright is a central illness in this group.

### Freelists of treatments

Having identified the most salient illnesses in the community, the next step was to find the most salient treatments for those illnesses, including fright. Local research assistants and I conducted free-listing interviews for remedies with every willing adult villager (N = 112, over 90%) in residence during the summer of 1998 [44]. We asked villagers to list all the bush medicines that treat each of the salient illnesses.

### Salience analysis of treatments

The responses for interviews on each of the salient illnesses, individuals' lists of treatments were tabulated using the salience method noted above. This analysis yielded an inventory of the most consensual treatments for the common illnesses that Dominicans treat with bush medicine. Top-scorers here are the herbal prescriptions that form the village's core pharmacopeia. This salience-finding process allows for discussion of the most shared treatments for fright, thereby eliminating treatments that may be unusual, idiosyncratic, or "noise" in a cultural sense.

### Plant recognition and use survey

Local research assistants and I used a structured survey with all willing village adults (N = 106) to appraise the community's knowledge and personal use of the most common medicinal plants (the 32 most salient medicinal plants that comprise the villages core pharmacopeia). This methodology was modeled on Berlin and Berlin's "traveling herbarium" technique [48,49]. To probe for informants phytotherapeutic knowledge, the Berlins used pressed, dried, mounted, plastic-sealed plant specimens, carried in a 3-ring binder. Instead of using real pressed plants, I used a traveling botanical photo album containing photographs of each plant growing *in situ *in the village. (Herbarium samples were collected, with help of key informants, for each of these plants; however, subjects saw only the photographs of live plants.) The survey that accompanied the botanical photo album asked for each plant:

1. Do you recognize the plant?

2. What do you call the plant? (name or names)

Next, for each of the eighteen salient illnesses, the informant was asked the following:

3. Do you use [this plant] to treat *illness 1 [e.g., fright]*?

4. What part of the plant do you use for *illness 1*?

5. Method of plant preparation for *illness 1*.

6. Duration of treatment for *illness 1*.

7. Have you used it for *illness 1*?

Because most questions were repeated for each of the 18 illnesses, each person's interview yielded 92 data points on 32 plants for a total of 2944 data points per subject. Informants generally hastened through these questions, which mostly required only yes/no responses, and interviews took between 45 minutes to 1.5 hours to complete. Because of the matter-of-fact nature and quick pace of this instrument, questions and responses about fright were not particularly personal, and so responses were neither sensitive nor emotional; neither were they detailed. However, they do provide a basic count.

There were three salient treatments for fright in this survey. Epidemiologic data for fright then comes from individuals' answers to question 7 above, which asks if the person has used the plant for fright. An affirmative answer to that question suggests that the subject has had fright, or at least has suffered from fright to the degree that he or she felt that treatment was necessary.

### Demographic variables

Subjects' sex, age, years of schooling, and how many children they had were recorded along with the freelist interviews. As a proxy for wealth, I use a measure of consumerism. This works well for Dominican villagers because they generally own their household goods outright, rather than through credit or debt. I measure consumerism by an inventory of purchased household goods (e.g., electricity, jambox, stove, telephone) collected with Rob Quinlan. The more purchased items a household has, the higher its consumerism score (item analysis yielded a set of scaled items that was unidimensional [measuring a single construct][39], see [50] for details). Every adult in a household shares the same consumerism score.

## Results

### Knowledge of fright

Fright is a universally recognized illness. When villagers freelist home-treated illnesses, fright falls in a middle-position of the most familiar illnesses, ranking 17^th ^of the 32 illnesses. Fright's salience score (using Smith's salience statistic [46]) was .205, compared to the most salient (worms) with a .523, and the least salient (toothache) with .003. The freelisting method elicits items that are so familiar that informants can recall them immediately by name. Freelists gauge *active *knowledge/vocabulary--items of psychological or cultural preeminence--and individuals may not list various items that they know [51]. Rather, salience of freelisted data is closely related to familiarity or regularity. For example, Dominicans take prophylactic worm treatments routinely (weekly to monthly) [52] and worms appeared as the most salient illness. The least salient illness, toothache, is also a matter of fairly common knowledge; however, toothaches occur sporadically and people only treat them as-needed. (Further, there is no ethnobotanical pain control that is as good as pharmaceutical analgesics according to most Bwa Mawegans. I suspect declining reliance on bush medicine for pain.). Fright's mid-way ranking amongst illnesses indicates that it is a domain of common knowledge. Indeed, in the illness-focused freelist on fright (conducted with almost every village adult) all adults recognized fright as an illness, and only 3% of adults could not recall the name of a fright treatment on the spot.

### Conceptions of fright

According to Dominican humoral theory, fright (the emotion) is freezing cold. Experiencing emotional fright sends the body into the coldest possible human humoral state. This condition occurs immediately upon experiencing the emotion, and the sudden onset may "shock" a person's system. During this shocking period, one's cold blood allegedly congeals, which slows a person down. Or, the cold blood may coagulate or freeze into a "mass" which can block blood flow and kill the sufferer suddenly with a stroke or infarction. The humorally cold body of a frightened person is at risk generally as the sufferer is then susceptible to other cold illnesses (e.g. respiratory illnesses).

Strong emotions not only chill the blood, but make it sour and caustic to the nerves. The initial cause of fright is an emotionally-charged cold humoral imbalance, but the frightened patient's caustic blood can cause him nerve damage which aggravates the fright and delays recovery. (Dominicans reckon that an excess of hard alcohol or processed drugs in the blood similarly damage nerves. Substance-damaged nerves, however, do not lead to distressed symptoms of fright, but rather to madness or dependence.)

The kinds of events that "frighten" a person include suffering a near-death experience, receiving news that a loved one is severely injured or dead, having a fight with someone close, or seeing a witch. Fright is hence one of the few illnesses that Bwa Mawegans attribute to both personalistic and naturalistic (*sensu *Foster [53]) causes, i.e. the emotions that produce the illness may have natural or supernatural origins.

Dominicans recognize two variants of fright illness which they categorize by their duration. There is a regular or short-term fright, usually called "fright," or occasionally distinguished as "short fright" variety; and there is a long-term or permanent "chronic fright." The variants are contrasted in table 1. Both frights are reckoned as physical (or, actually, whole mind-body) manifestations of emotional frights or traumas. Both include recurrent recollections of the traumatic events, loss of concentration, and frequent outbursts of anger or grief. The short fright, arises from a single occurrence, and includes a period of persistent arousal and hypersensitivity. In contrast, the chronic form of fright is caused by repeated stressful occurrences and, whereas the short-term sufferer is constantly tense and sensitive, the chronic suffer becomes persistently dull and cheerless.

### Treatments for fright

**Figure 1:**
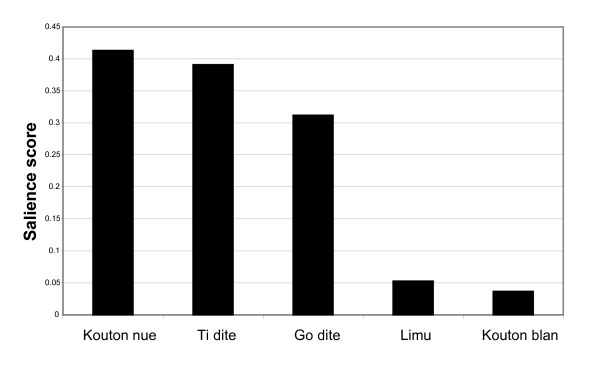
**Salience of freelisted fright treatments**. This chart shows the relative salience of listed fright treatments using their *Kwéyòl *common names. *Kouton nue *is *Gossypium barbadense *L., red leaf color variety. *Ti dite *is *Lippia micromera *Schauer. *Go dite *is *Plectranthus amboinicus *Sprengel. *Limu *(or *limu du mer*), is "sea moss" which grows on seaside rocks (unidentified), *Kouton blan *is again *Gossypium barbadense *L., but the green leaf color variety, locally recognized as a separate plant.

The salience of freelisted fright treatments appears below in figure 1. Three plants were particularly salient fright remedies (Those were *Gossypium barbadense *L. (with the Dominican common name *kouton nué*), *Lippia micromera *Schauer (*ti dité *in Dominica), and, *Plectranthus *[Coleus] *amboinicus *[Loureiro] Sprengel (*go dité *in Dominica). None of these is native to the island, though they are naturalized there. The most salient treatment, *G. barbadense *L. may have made it to Dominica before Europeans and Africans.

Locals view these plants as humorally hot, and thus able to counterbalance the cold impact of a frightful emotion on a sufferer's body. Dominicans make an infusion or "bush tea" with each of these medicinal plants. Because Dominicans acknowledge the plants as hot or warming, a bush tea made with one of these plants is considered humorally hot, or warming to the body's system, whether drunk warm or completely cooled. In local parlance, the hot tea melts frozen congealed blood back to normal. Nevertheless, the body will continue to re-cool itself for as long as the frightening emotions last, which may be many months (as with an individual in mourning, for example). If a person has a short-term scare, the course of his fright illness will likely be rapid, and a few cups of bush tea over several days may suffice to restore the person's balance. Normally though, fright requires a long period of coping, and therefore a long period in which one's body tends toward a humorally cold state. People living through a fright illness use these teas regularly, generally alternating from one to another.

In open-ended interviews, Dominicans also note that time, prayer and exercise are necessary in the course of therapy for fright. Though Bwa Mawegans do not necessarily consider prayer and exercise (or physical work) medicine, people view these activities as essential to good living and to the healing process, particularly for fright recovery.

### Epidemiology

**Figure 2:**
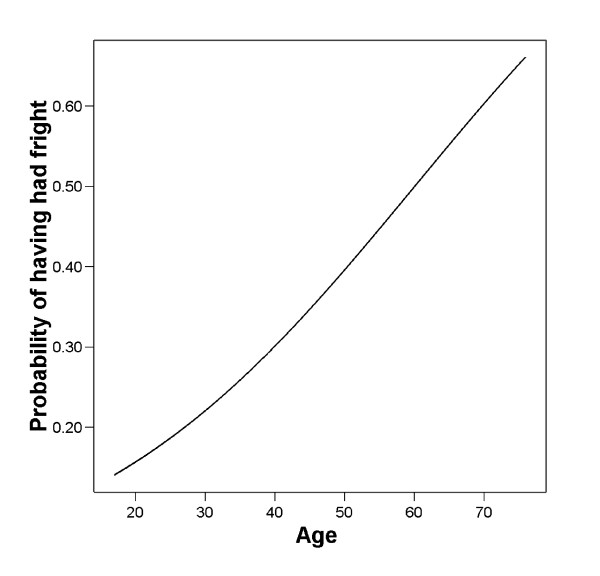
**Relationship between fright and age**.

Amongst a battery of other questions, I surveyed village adults regarding whether each of the salient fright plants (*kouton nué, ti dité *and *go dité*) was a local fright remedy, and specifically, whether the individual had used each of the fright herbals to treat fright. Twenty-eight of the 103 (27%) adults had treated themselves for fright. In a logistic regression, I regressed individual presence or absence of having fright with demographic data on respondent's age, education level, gender, parental status and wealth measured in consumer goods (see table 2). Controlling for the other factors, only age turned out to be a good predictor of whether one had had fright (see figure 2). Parenthood marginally reduces one's risk of fright. Thus, with every year one lives a person appears to increase the probability of becoming "frightened" enough to require medication.

**Table 2:** Logistic regression of fright on to demographic variables

	B	df	Significance
Age	0.0673	1	0.0046**

High School	-1.4092	1	0.245

Sex (F = 1)	0.03741	1	0.565

Parenthood (f = 1)	-1.4615	1	0.0859*

Wealth	0.0402	1	0.6865

	**N**	**Percent**	

Included in analysis	78	49.1	
Missing cases	81	50.9	
Total	159	100	

## Discussion

### Ethnophysiology

Dominicans ascribe to a version of the hot/cold humoral theory, which has been documented throughout the New World, particularly in Latin America (for an overview, see [16]). In the hot/cold humoral system, people group mental and physical states, plants, and animals into "hot" and "cold" categories. The foundation of rural Dominican humoral theory is that humans are made of meat. Locals equate the behavior of human flesh and fluids to that of the meat and gravy in their daily stewpot, which becomes thin or supple when warm and thick or hard when cool. Thus, if temperature, food/drink or emotions create too much cold inside a person's body, his bodily fluids and tissues presumably thicken or harden. Hard tissues or thick body fluids are the perceived etiology of a cold illness. Conversely, when temperature, food/drink or emotions result in too much bodily heat, a person's insides soften and thin, or (in extreme cases) cook.

Fright is a freezing-cold feeling that results in the coldest humoral state. Because fright happens suddenly, the transition from a normally warm body-state to a cold one "shocks" ones system. There are other ways to experience a cold shock--bathing in cold water too soon after working in the heat, for example--but fright is the gravest kind of shock.

Fright is an emotional illness--an emotional alarm response to a trauma sets the illness off, but the symptoms of fright are also emotional and include anger, grief, nervousness and sadness. The kinds of events that cause fright are generally involuntary emergencies, such as the sickness or death of a loved one. Occasionally though, an assumed run-in with a witch will frighten a victim into illness, as such an experience is viewed as a brush with death, and implies future risk of evil-doings.

Bwa Mawego residents believe that local witches have learned how to change their form, and do evil. People are not born with this ability. Anyone, male or female, young or old, could learn witchcraft. No one knows for sure who the witches in the village are, but any adult is a potential suspect as one of four types of shape-shifting witches. Two of the witch types enter the houses of their enemies and the people they envy, and they suck the family's blood. Seeing such a lethal witch would probably propel a person into fright, though the witch's express purpose is blood-sucking. Two other types of lesser witch exist solely to shock fellow villagers into fright by turning into startling, creepy animals and strange people (see [29]).

Fright is described as making one's blood chill and thicken, or even freeze into clots or "masses." If a mass blocks circulation to the brain or heart, a person could drop dead suddenly. Otherwise, cold blood might just slow a person down. The cold state opens a fright sufferer to comorbidity with a host of other illnesses reckoned as humorally cold (noted below).

### Short vs. chronic fright (etiology and symptoms)

Dominicans recognize two kinds of fright illness, which differ in their chronicity. Both are long-term conditions, lasting at least several months. The "short" variety, however, subsides eventually, while the other variety, "chronic fright" is permanent and "can kill." Diagnosing which variety of fright a person has is, to some degree, a matter of time, with chronic fright as the default diagnosis. Dominicans nonetheless know which fright variety to expect by the nature of the emotional traumas believed to cause the illness. The shorter-term variety occurs in the wake of a single traumatic event, such as a near-death experience, a fight, or the death of a loved one. The "chronic" or permanent variety is the cumulative effect of successive stressors--"trials and inequities" that "scrub away a persons nerves" until the frightened person is no longer fully functional.

One Dominican grandmother explained that "short fright" begins with a shocking event that leaves a person stunned.

"Let's say you loose a family member: You get frightened. You are just in one place and can't move. Then, some people get the sickness after. Those people who had a big shock stay frightened for maybe a year, maybe more...But most learn to cope and the fright cures. People drink bush (herbal) tea to keep the fright down."

In contrast, she noted that

"Those people that have mental patients, drug people, abusive husbands, bad neighbors--troubles that are repeating--they can not get over the fright. The bush (herbal medicines) help, but not enough...Once the fright has been there for a few years already, you know that this person's fright is the chronic one that cannot cure."

She added that chronic fright sometimes progresses until finally "the frightened person can not get out of bed, and even though you try to feed them, they die there."

In the short-term variety, symptoms of a frightened person include frequent recollections or dreams about a traumatic event, difficulty concentrating, persistent anxiety or arousal, hypersensitivity (including exaggerated startle responses) and outbursts of anger or grief. The characteristics that Dominicans associate with "fright" are similar to symptoms that Hispanic Caribbeans (e.g. [22]) and other Latin Americans (e.g[23]) associate with *nervios*, and highly comparable to the set of symptoms that biomedicine associates with Posttraumatic Stress Disorder--which are also precipitated by an emotional trauma [54].

In permanent or "chronic" fright, the sufferer continues with most of the "short fright" symptoms, except that he is no longer persistently aroused or hypersensitive, but rather, becomes "dull," frequently sad, tired, and "not really stupid, but foolish--like the brain's getting lazy." Some people live with chronic fright into old age. But, in extreme cases, the frightened person becomes permanently depressed (without even happy moments), cannot eat, cannot sleep or sleeps all the time, and has some psychotic events. These people will die of fright. Chronic fright shares traits with *nervios *([55], see also [24]) as well as *susto *[8,19] and perhaps best matches Western psychiatric symptoms of major depression [54].

Dominican thinking is that almost anyone, if bombarded with enough stress, might develop a fright illness, but the severity of the illness, and indeed whether one becomes ill at all, depends on the "strength" of one's God-given nerves. People born with "weak" nerves are more nervous and irritable, and are said to be at risk for fright. Similarly, if a Puerto Rican is said to *ser nervioso *(be a nervous person) he is vulnerable to various attacks and chronic states of *nervios *[22]. In Dominica, an individual's vulnerability is always, to my knowledge, noted in retrospect, as a *post hoc *explanation after the onset of fright. It is not normal to acknowledge a healthy person (or self-identify) as having weak nerves or being at risk, and no preventative measures exist for these people.

In addition to the above symptoms, both long and short-term frights include or are associated with secondary problems that people attribute to the constant cold state caused by the fright. Cold, in addition to thickening blood, affects the viscosity of "white" body fluids (mucus, breast milk and semen). Frightened people get colds more often or "constantly." Fright might "freeze" or thicken a woman's breast milk so that she develops mastitis or otherwise can no longer nurse (cf [2]). Men who have suffered from "fright" can likewise experience impotence attributed to frozen sperm. Cold can collect around the joints and stiffen them; resulting in the kind of rheumatism blamed on fright, called "a-fright-is" or "afritis" ("afritis" is also how to pronounce "arthritis" in Dominican English Creole, in which th in Standard English shifts to f after a vowel). These comorbid conditions with fright in the Caribbean resemble Baer and Bustillo's findings that Mexican and Mexican-Americans mothers in Southwest Florida associate physical symptoms (diarrhea, vomiting, fever, weight loss) with their young children's *susto *even though susto is a social and emotional illness [56].

### Remedies

When one talks to Dominicans about fright remedies or "cures," (their preferred word), people always mention their (herbal treatments). Using "bush medicines" is a ubiquitous village response to physical and emotional woes, and fright is no exception. I discuss herbal medicines later. I first discuss villagers' non-medicinal responses, as these views and practices are the setting for medicine use.

Dominicans note that recovering from fright will inevitably take some time and requires prayer. Interviews indicated that the time range for "short fright" is from two to fourteen months--possibly up to two years. The amount of time to heal is said to depend on the individual's attitude, his social support, the God-given strength of his nerves, and the nature of the frightful event. Regardless, Dominicans believe that if one is patient, trusts in Jah (God), and prays for help, one increases one's rate and chance of recovery.

Dominica is a traditionally Roman Catholic country and approximately 77% of Bwa Mawego residents are at least nominally Catholic, while 15% are evangelical Protestants, and many maintain independent Rastafarian beliefs alone or alongside their church-based ones. Prayer is a traditional coping mechanism in Bwa Mawego and a common response to any problem. Prayer is a means of dealing with psychosocial syndromes in numerous-if not most-societies (e.g. for depression among Caribbean immigrants in UK [57], for stress among South African township black women [58], for *scantu *(fright) in Sicily [59], for alcohol use, major depression, and PTSD among Navajo [60], and throughout Latin America for *susto *[19] and *nervios *[23]. The largely positive role of prayer and religiosity in mental health is well established (for a review see [61]).

Some Dominicans mention that exercise is the best treatment or "the doctor" for fright or any kind of stress, and that one should "burst a good sweat" to "warm out the fright." This advice is concurrent with biomedical thinking that alarm reactions trigger endocrine responses which prepare bodies to cope with threats through "fight or flight" (i.e., exercise) and, hence, physical exercise effectively "ventilates" the stress response [62]. Aerobic exercise has demonstrated antidepressant, antipanic, antianxiety effects (for a review see [63]), and, individuals' general activity levels associate inversely with stress pathologies [64]. About a third of my consultants specifically mentioned exercise as a treatment. Perhaps most individuals omitted non-pharmacological responses in discussions with me (because of either what they considered, or thought I considered, a "treatment"). Or, it may be that, exercise is a given for most people in this community, and therefore not considered worth mentioning. Because of the steep, rugged terrain, and dependence on walking, carrying loads, and subsistence gardening, a Dominican villager generally "bursts a sweat" several times a day if he is able-bodied enough to leave one's home at all. As Dominican villagers become feebler with age their physical tasks become less strenuous, i.e., less sweaty. Rather than hauling things they stay home and work more on stationary tasks such as peeling food, cleaning and working in the house garden. The relative lack of hard exercise may make elderly Dominicans more susceptible to fright.

Along with the above strategies, most Dominicans use bush medicines to treat fright. Medications that Bwa Mawegans use for fright are humorally warm. They help to "melt" the chilled blood masses. Bwa Mawegans say that a person often feels soothed after just one dose (usually a cup of "bush tea" [herbal infusion]) of a fright treatment. However, the cold in the blood may start to build up again after a few hours. Someone suffering from fright may "cure" within a couple days of his or her shock. However, if one's trauma was particularly horrible, or if one has particularly weak nerves to begin with, he might suffer from fright--and continue taking bush treatments for it--for up to two years, and sometimes sporadically after that. People with lasting cases of fright typically vary their herbal treatments every few days. I review the salient herbal treatments and their respective literatures below.

### *Kouton nué* -* Gossypium barbadense* L

#### Voucher accession number UMO-186416, University of Missouri herbarium

In Bwa Mawego, Dominica, the number one phytotherapy for fright is the plant villagers call *kouton nué *in both French Creole and Creole English, though occasionally people use the English name "black cotton." This species, *Gossypium barbadense *L. (Malvaceae) was Bwa Mawego's most salient treatment for fright, listed by 48% of informants. *Gossypium barbadense *is a native to tropical Northwestern South America and Caribbean Island and Central American forms of the species derive from a species diffusion path across northern South America east of the Andes [65] It is a morphologically diverse species consisting of a wide range of wild (or feral) types[65]. *G. barbadense *was one of the first American cultivates, farmed in the Andes by about 10,000 years ago [66]. It remains a commercially important cotton species that is grown in many regions of the world[65]. Common names for *Gossypium barbadense *cultivates include Extra Long Staple, Pima, South American, Creole, Sea Island, Egyptian cottons. The species accounts for about 5% of the world's cotton market and is prized for having the longest, highest quality fiber [67]. In Dominica plants grow cultivated and as escapes [68].

Bwa Mawegans do not trim *G. barbadense *trees and they grow to around three meters tall in the village. The large, usually red-tinged leaves (40 cm) are alternate, on long petioles (at least 10 cm), and have five triangular lobes. The inflorescence is ivory to yellowish, and the cotton around its seeds is grayish, and darker gray on the red-leaved trees. Though the single species has a range of coloration, Dominicans have separate common names for the red-tinged plants with dark fiber, and green-leaved plants with light fiber: *kouton nué *(black cotton) and *kouton blan *(white cotton). In freelists, kouton nue was a much more frequent response, though some individuals specifically mentioned *kouton blan *in addition to or instead of *kouton nué*. There are five mature red-tinged trees growing near people's houses in the village, and I only know of one large fully-green-leaved tree. There are also several smaller, less visible shrubs in various home gardens.

If a Bwa Mawegan wants to make *kouton nué *tea, he asks one of his neighbors who has a *kouton nué *tree for some leaf. This is a small request, as the tree's leaves are so large. One can make at least three servings by boiling one lobe from one leaf (torn up) for around three minutes. Villagers with fright drink a cup of this tea once or twice a day until their fright subsides. As with all fright treatments, villagers say that bush tea made with *kouton nué *relaxes a person and "warms" his "frozen blood."

Ethnographic information on the medicinal use of this plant, aside from the cotton fiber, is relatively scanty, but most uses relate to relieving pain or treating coughs and respiratory trouble. In North America, the Alabama and Koasati Indians used the roots of *Gossypium *ssp. to make a tea to ease childbirth (Moerman 1998). A decoction from the bark of cotton roots was an official U.S. drug used from 1863 to 1950 to stimulate menstrual flow and aid contractions during labor. Northern Peruvians use *G. barbadense *topically for wounds (Bussmann and Douglas 2007), and Jamaicans use it for hemorrhoids [5]. Yucatan Maya[69] and people of St. Kitts[70] drip juice of the flower bud into the ear for earaches. Venezuelans (Chiossone V. 1938), Trinidadians [71], Jamaicans [72], and people of St.Kitts and Nevis [70] drink an infusion of *G. barbadense *leaves and flowers for bronchial and pulmonary problems, and colds and flues. Cubans boil the seeds to make a decoction for bronchial trouble [73]. South Carolinians [74] and ancient Maya [69] used the gossypium roots for asthma. Maya of Chunhuhub (Quintana Roo) Mexico use the *G. hirsutum *plant for snakebites, and grind the seeds for a headache poultice (Anderson Eugene N, with Cauich JC et al. 2003). Trinidadians favor a treatment of cotton seeds (of various species) for deworming dogs [75]. Several peoples across the Guiana shield use *Gossypium *ssp. leaves medicinally (DeFilipps, Maina et al. 2004). They are a backache treatment throughout the area. French Guianese and several native Guiana groups (Caribs and Arawaks in Surinam; Palikur and Wayapi in French Guiana, Patamona in Guyana) boil *G. barbadense *leaves to treat high blood pressure, and pain, and apply macerated leaves to control itching. In the Guianas, *Gossypium *treats pain and stress in some accord with the Dominican use for fright.

In comparison to the abundant laboratory research on agricultural aspects of cotton, pharmacological research pales. We know, however, that Gossypol, produced especially in the seeds in *Gossypium *ssp. is highly toxic to fungi that are pathogenic to animals (Mace, R.D. Stipanovic et al. 1993); and gossypol is particularly effective against the trypanosomosis diseases including African Sleeping Sickness and South American Chagas Disease (Woerdenbag 1993). Gossypol is also cytotoxic to tumor cells (Woerdenbag 1993). Gossypol functions as a contraceptive for men--it is antispermatogenic, reducing total sperm count and sperm motility and velocity (Coutinho EM, Segal SJ et al. 1985). *Gossypium *leaves contain red and green pigments (gossypurin and gossyverdurin) which have similar chemical structures to gossypol in the seeds (Waller, Zaveveld et al. 1985). Infertility may be an unwanted side affect of gossypium medications, however (Lewis, Elvin-Lewis et al. 2003).

### *Ti dité* - *Lippia micromera* Schauer

#### Vouchers UMO-186354, UMO-186385, University of Missouri herbarium

The second most salient Dominican herb for fright is *Lippia micromera *Schauer (Verbenaceae), known locally as *ti dité*. Informants mentioned *ti dité *in 45% of the fright freelists. Bwa Mawego residents call *Lippia micromera *"ti dité*" *when speaking both English and French Creoles, and, rarely, "small tine" (pronounced with an N) in English. Elsewhere, common names for this species include "false thyme," "Spanish thyme," and *oregano del país *[76].

**Figure 3:**
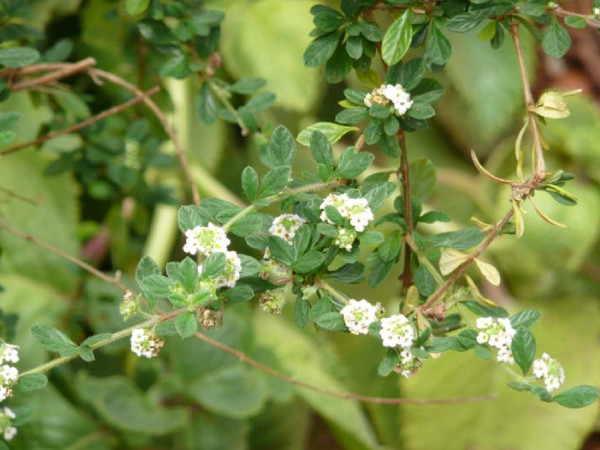
**Close-up view of Dominican *ti dité, Lippia micromera *Schauer**.

**Figure 4:**
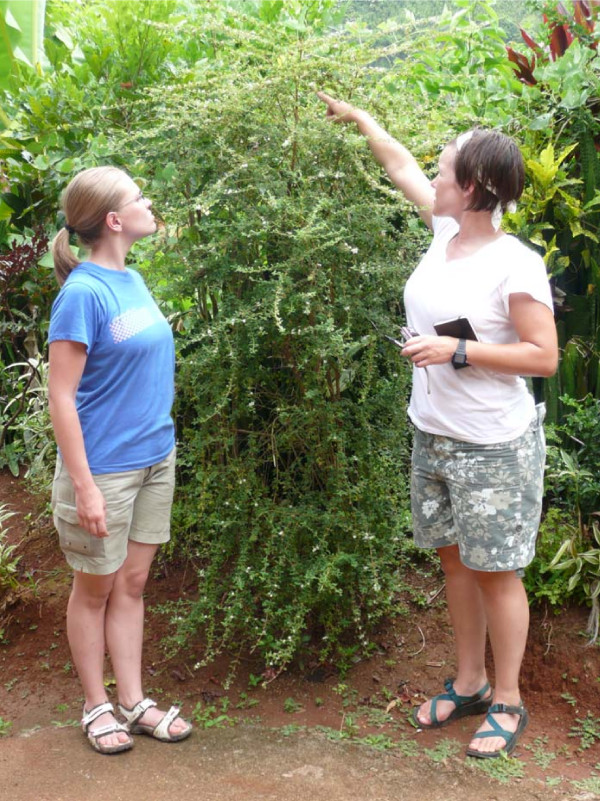
**Full-plant view of a larger Dominican *ti dité, Lippia micromera *Schauer, plant with Washington State University students Christie Stordeur (left) and Sarah Council, 5'3" (on right)**.

Ti dité, *L. micromera*, is a thyme-scented shrub that is often about a meter high locally, but reaches nearly two meters (see figure 3). It has thin, woody stems bearing opposite pairs of small (<12 mm) oval leaves and inflorescences that are tiny, white, tubular flowers with yellow throats growing in heads (figure 4).

Like the first humans to inhabit Dominica [77], *L. micromera *is native to the northern, Caribbean coast of South America--i.e., Columbia, Venezuela and the Guianas, and possibly to Trinidad and Margarita Island [76]. *L. micromera *occurs elsewhere in Dominica [68,78] and is cultivated in home gardens throughout the Caribbean [76]. It is a seasoning in Northern South America, Central America and the Caribbean that people especially tend to eat with meat in soup and gravy [79].

In Bwa Mawego, *ti dité *is common for seasoning and medicine and one of the village's most familiar herbs. Practically every household tends a *L. micromera *shrub in its home-garden. Locals report that *ti dité *bush tea is calming and warms the blood.

The literature contains various mentions of tropical peoples' medicinal use of *Lippia *ssp. (see [80] for genus review); however, there are few ethnographic reports for *L. micromera *specifically. People of San Felipe, Yaracuy State, Venezuela use *L. micromera *for upset stomach [81]. In Montserrat and Trinidad people use a *L. micromera *infusion for colds [71,82], and also for influenza in Trinidad [71], and sore throat in Monserrat [82]. It is an antispasmodic across Venezuela [83].

African and American tropical peoples most usually use Lippia species to treat respiratory disorders [80], for stomach problems and as sedatives ([80] and [84] for review of *L. alba *in South America). For example, Jamaicans use *L. alba *for insomnia [5], and Brazilians use *L*. *alba *and *L*. *geminata *as sedatives [85,86], and Nigerians likewise drink an infusion of *L*. *geminate *as a sedative and relaxing remedy [87].

Haitians use a plant with the common name *dité *(c.f. *ti dité *in Dominica), for what the researchers identify as "nerves"[88], which may relate to a similar or identical condition as fright. Though this research identifies Haitian *dité *as *Thymus vulgaris*, it may even be *L. micromera *('false thyme") rather than (*T. vulgaris*) ("thyme") as this misidentification is common in my experience. At any rate, thymol is the main constituent in both *T. vulgaris *and *L. micromera*, and the effects of the two plants should thus be somewhat comparable. European use of thyme tracks perfectly with the Caribbean use of *ti dité *bush tea for fright. For example, in England, the "most important reason for drinking thyme tea has been to calm the nerves: the plant is a well-known sedative ([89]:224)." Further, Scots and English of Suffolk County used it to prevent bad dreams [89].

Pharmacologically, *L. micromera *demonstrates bioactivity in brine shrimp assays [90], and as extracted essential oil [91], and in vapor phase *L. micromera *shows highly potent antimicrobial activity against Gram-negative and gram-positive bacteria, molds and yeast [92]. Bacterial strains are highly sensitive to *L. micromera *oil, and fungi and yeast strains are even more sensitive than bacteria to the action of the oil [91].

*L. micromera*'s leaf oil is high in cavacrol (42.2%)[93]. Cavacrol performs diverse pharmacological activities (see [94]including inhibiting gastrointestinal spasms and contractions in rats [94]and rendering bacteria non-infective (by affecting flagellin such that cells become nonmotile and unable to adhere to epithelial cells) [95].

The painkilling ability of *L. micromera *remains uninvestigated, however oregano oils- which, like lippia oils are very high in cavacrol- demonstrate analgesic activity [96], and *Lippia adoensis *is analgesic for mice [97].

*L. micromera*'s essential oil (as opposed to leaf oil only, above) is abundant in thymol (33.7%) [91]. Thyme oil (whose principle constituent is thymol, like *L. micromera*'s essential oil) displays spasmolytic muscle-relaxant, as well as antibacterial, antimycotic and antioxidative properties, and is among the world's top ten essential oils [98]. Thyme oil was formerly prescribed for dyspepsia, dysmenorrhea, headache, to relieve "hysteria" and "exhausting diseases", and as a soporific [99], and biomedicine continues to use the thymol, in various common topical ointments and antiseptic solutions (e.g. Vicks^® ^and Listerine^®^). It is a prescription for intestinal parasites, particularly hookworm [100]. Because thymol can irritates gastric mucosa, pharmaceuticals largely avoid its internal use; yet thymol remains an official United States Pharmacopeia (USP) drug [101]. Most interesting in terms of treating fright, thymol, the principal constituent in *L. micromera*, inhibits neurotransmission in the central nervous system (has GABAergic activity) [102] and thus regulates excitability and has general anesthetic properties (similar to propofol) [103].

### Go dité - *Plectranthus amboinicus *Sprengel

#### University of Missouri herbarium vouchers UMO-185582 and UMO-186388

**Figure 5:**
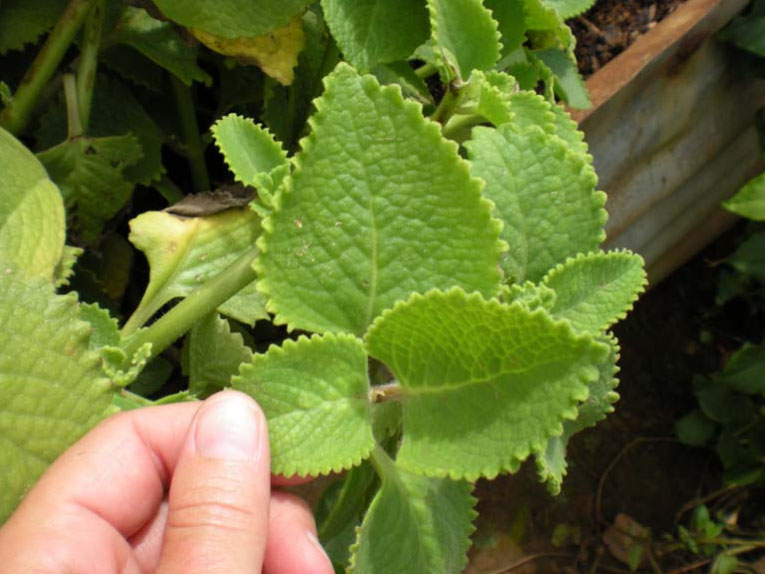
**Close-up view of Dominican *go dité*, *Plectranthus *[Coleus] *amboinicus *[Loureiro] Sprengel (photo by Sarah Council)**.

The third most salient treatment for fright is *Plectranthus *[Coleus] *amboinicus *[Loureiro] Sprengel (Lamiaceae), which Dominicans call *go dité *and *go djai*, whether speaking English or French Creole (figure 5). (The species is widely used in tropical regions and outside of Dominica has numerous English common names including Cuban oregano, Mexican mint, Indian borage, Spanish thyme, and French thyme.) In freelists, Dominicans listed *go dite/go djai *one time more than *ti dité *(38 mentions, or 46% of informants), however these mentions fell further toward the ends of individuals' freelists making the plant slightly less salient statistically (*go dité (P. amboinicus) *silence score is .31 and *ti dité *(*L.micromera*) has a .39, a 7.9% difference). *Plectranthus amboinicus *is a native of the East Indies that has become naturalized in some of the West Indies [104]. It is a semi-succulent herb that reaches a height of 50 cm. It has opposite, deeply veined leaves 3-6 cm wide by 4-8 cm long with obvious hair and serrated edges. The square stems are rounded. The flower is violet. I have only observed this plant growing in village home gardens, where it is an essential household herb.

In addition to its medicinal role, *go dité *is an everyday cooking herb in Dominica, (as elsewhere in the middle Americas). Nearly every family has some of this plant growing near their home. Dominican villagers consume *go dité *almost every day in very dilute amounts (one leaf per 4 liters of stew). Medicinally, it is a hot herb that Bwa Mawegans always ingest in the form of tea. Besides using *go dité *for fright, some Dominican women use it to induce menstruation and labor, throughout labor, and after childbirth. To make a medicinal bush tea, villagers use two average stalks (containing around 24 leaves) to one liter of water. Some people make a decoction, boiling the tea for a couple of minutes, while others make an infusion by steeping *go dité *for several minutes. Villagers say that one can feel the calming, "melting" effects of *go dité *"bush tea" immediately. One cup of tea may be enough to cure a person of an acute attack of fright. A chronic fright sufferer takes one cup a day, or a few cups a week, usually alternating with the abovementioned herbs.

This species is used medicinally elsewhere (For a review of *Plecthranthus *ethnobotany see [105]) Peoples of North, East and Central Africa, Asia, South America, the Caribbean, and the Pacific use it for digestive problems and skin conditions including burns, wounds and allergies [105]. Like some Dominicans [106], Brazilians in Bahia use ground *P. amboinicus *leaves topically to treat skin ulcers [107]. In the Amazon and India the leaves of *P. amboinicus *treat urinary diseases [107-109]. Asians and South Americans use it for fevers [105], and Cubans use it as a bronchodilator and general cold and respiratory remedy [73]. Schultes and Hoffman find that a related species (*Plectranthus scutellarioides *(L.) R. Br. [as *C.blumei*]) has psychoactive properties and has magico-religious significance for the Mazatec in Mexico as "divinatory" [110]. Most concurrent with its Dominican use as a fright treatment are other uses of *P. amboinicus *as a relaxant or antispasmotic, and to treat pain. Cubans prescribe it for epilepsy and convulsions [111,112] and for earaches [73]. Kenyans use it for stiff necks and backaches [113]. Asprey and Thornton report historic uses of *P. amboinicus *in India (by its former *Coleus aromaticus*) to treat cronic cough, asthma, epilepsy and other convulsions, and also note that it had an intoxicating effect([72] and [114,115] in [72]). Trinidadians and Jamaicans also use it for coughs, asthma, epilepsy and convulsions [116].

So far in pharmacological laboratory screening, *P. amboinicus *shows antimicrobial activity [112,117]. It demonstrates antiviral activity against vesicular stomatitis [118], *Herpes simplex *virus-1 [119], and it inhibits HIV activity [120]. A decoction from *P. amboinicus *leaves demonstrates anti-epileptic effects [121], and, in experiments on rats, the decoction indicates simultaneous actions similar to those of tricyclic antidepressants (TCAs)[122].

### Epidemiology of fright

Rates of fright are fairly low. The frequency of fright is such that during any given field season that I have been in this village, there may be community knowledge of one to three individuals (out of about 130 adults) suffering fright -- sometimes nobody is frightened, or sometimes a family of ten or so may be. The irregular and fairly uncommon nature of the illness makes it hard to asses at the community level.

Further, finding about the epidemiology of fright in Dominica is not a simple task because people do not like to talk about their own fright experiences. Dominican culture has a strong Christian ethic of "neighbor loving," such that if one resident is publicly ill or troubled, his neighbors must try to help the sufferer. And, sufferers do receive plenty of outside attention. And yet, from the sufferer's view, it is his social responsibility to keep unpleasantness to himself, or within his immediate family, so as not to oblige his neighbors, who have other responsibilities. Further, should a neighbor become frightened or worried for a suffer, the neighbor may himself develop a cold humoral imbalance. Dominicans are stoical about their problems and outright complaining is a social infraction. This stoicism resembles Tzotzil Maya obligations to control negative emotions because emotions are linked to physical sickness, such that emotional management is encouraged at both the individual and social levels [123] Neighboring Latin cultures may offer a different sort of social control. Less stoic, and more prone toward martyrdom, they use the cultural idiom of *nervios *and (*susto *less so) as a cry for help and a means to adjust social obligations to a more manageable level (e.g. [25]). Dominican stoicism seems to preclude using fright to realign social obligations. There is, nevertheless, no stigma to suffering fright, particularly the short-term variety.

Recall of fright is nevertheless unreliable, perhaps because it is trauma-related. Several villagers in the throws of fright have discussed their cases with me. But on later occasions, when the suffering was over, those individuals told me that they had not had fright. If I further probed, "Didn't you have fright after so-and-so died?" they told me, "Well, no, not *really*." Given that few people have (or share) fright at one time, and that people are uncomfortable recalling *past *fright experiences, finding the overall incidence and risk factors of fright is difficult.

While locals are often uncomfortable discussing personal fright experiences, reporting use of medicinal plants, used only to "melt" fright-caused clots, is not sensitive or problematic. As part of a larger medicinal plant recognition and use survey, I asked the vast majority of village adults if they recognized photos of the village's most common medicinal plants. I talked to 103 villagers specifically about all three of the salient fright plants. Among numerous other questions, I surveyed these interviewees as to whether each of the plants (*kouton nué, ti dité *and *go dité*) was a local fright treatment, and specifically, whether the individual had used each fright plant to treat fright.

In the survey interview schedule, which used the travelling botanical photo album stimulus, *kouton nue*, was the first fright treatment probed, and was the nineteenth herb on the schedule, which largely requires yes/no responses. Interviewees were likely thinking more mechanically in this context than in other contexts in which I broached the topic of their fright experience. Twenty-eight of the 103 (27%) adults had treated themselves for fright.

I put that data into a GLM logistic regression with demographic data on respondent's age, education level, gender, parental status and wealth measured in consumer goods. Controlling for the other factors, only age turned out to be a good predictor of whether one had had fright. Parenthood marginally reduces one's risk of fright. Thus, with every year one lives, a person appears to increase the probability of becoming "frightened" by something.

## Conclusions

A large corpus of research documents that stressful life events place one at higher risk for a range of psychological and physical disorders [124]. Particularly relevant to fright illness might be the association between traumatic events and occurrence of various anxiety disorders such as PTSD [125], and mood disorders such as major depression [126,127]. Caribbean ethnomedical theory also views fright sufferers at a risk for other humorally cold illnesses because of their cold blood. Common humorally cold illnesses include respiratory illnesses (asthma, cough, cold) and arthritis. Again, biomedical research corroborates in non-Caribbean populations that stress generally compromises the human immune system [128], and that psychosocial stress specifically associates with asthma [129], respiratory viruses [129], pain in osteoarthritis sufferers [130], and inflammation with rheumatoid arthritis [131]. To this extent, the symptoms and correlates of Caribbean fright concur with a cosmopolitan medical view of stress, *though the local idioms and explanatory models surrounding fright are Caribbean-culture-bound*.

Though getting fright is out of a sufferer's control, sufferers may actively affect their rates of healing by restoring balance to their systems if their life circumstances allow for it. Restoration involves (1) taking appropriate hot bush medicines, (2) stabilizing ones emotions and spiritual well-being through faith and prayer, and (3) getting exercise through continuing with their normal work.

A review of extant ethnopharmacological literature on each of the salient fright plants, suggests that the herbs may possess psychoactive medicinal properties that may help fright sufferers. Though some of the plants are well researched, research on the plants' psychoactivity is incipient and rare. This is par for the course as, aside from a large ethnopharmacological literature on hallucinogens, literature on phytotherapies for neurological and psychological ailments is scarce, as is pharmacognosy research on the plants (but see exceptions [132,133]).

It is ethnomedically interesting that the majority of the ethnopharmacognosy research and findings on the salient Dominican fright treatments, and indeed most medicinal mentions of these species in the literature, point towards these plants as cough or respiratory treatments, pain medications, and antibiotics and antivirals. Superficially, these uses might appear as "noise" when regarding the plants' use for fright as a psychiatric condition. However, Dominicans do not subscribe to a Western Cartesian split of mind and body. Despite mental symptoms, Dominicans view fright as a body (blood and nerve-based) illness. Baer and Bustillo conclude that dismissing psychosocial folk illnesses as non-medical concerns may be problematic because physical or comorbid symptoms associated with the folk illness may themselves be dangerous and benefit from biomedical treatment [56]. As mentioned above, fright's prolonged humorally cold body state puts fright sufferers at risk for other cold illnesses. The most common Dominican cold illnesses (besides fright) -- head colds, cough, asthma and arthritis-- all correlate with stress. Further, these illnesses are well served by medications that improve respiratory problems, viral and bacterial infections and pain. If these phytotherapies also offer fright sufferers relaxant and other neurologically therapeutic properties, all the better. From the standpoint of Caribbean ethnophysiology of fright as a cold mind-body condition (as indeed from the stance of psychoneuroendocrinology) each of these bush medicines addresses some, if not all, of the problems associated with fright.

Residents of Bwa Mawego report that *Gossypium barbadense, Lippia micromera *and, *Plectranthus amboinicus *are effective for fright, and the plants' continued use supports positive outcomes for locals. In ethnomedical ethnopharmacology, there is nevertheless much more to a medicine than the chemicals it possesses. Medicines have cultural and symbolic meanings that play into the power of their healing, even affecting the drug's biological effect on the human body [134]. Cultural beliefs about medicines can have additive effects for placebos and non-placebos alike. Moerman [135] terms this medical augmentation through cognition, the 'meaning effect,' which is like the 'placebo effect' but with emphasis on the symbolic value of medications. Plants with cultural salience have this kind of shared meaning to a local population. Thus, computing salience values for individual treatments within an illness domain not only indicates those species of the greatest common knowledge (which associates with likelihood of biological efficacy [136,137], but reveals plants that are most culturally or symbolically important in association with a specific illness. Particularly with a stress-illness like fright, the symbolic healing aspect of using these three salient, familiar, traditional treatments likely offers psychoneuroimmune (i.e. mind-body) benefits that intensify the plants' palliative value in addition to their stand-alone bioactive properties.

## Competing interests

The author declares that she has no competing interests.

## Authors' contributions

All research herein was conceived of, conducted or directed by, and authored by Marsha Quinlan.
